# Dielectrophoresis-Based Discrimination of Bacteria at the Strain Level Based on Their Surface Properties

**DOI:** 10.1371/journal.pone.0076751

**Published:** 2013-10-16

**Authors:** William A. Braff, Dana Willner, Philip Hugenholtz, Korneel Rabaey, Cullen R. Buie

**Affiliations:** 1 Department of Mechanical Engineering, Massachusetts Institute of Technology, Cambridge, Massachusetts, United States of America; 2 Australian Centre for Ecogenomics, School of Chemistry and Molecular Biosciences, The University of Queensland, Brisbane, Australia; 3 Advanced Water Management Centre, The University of Queensland, Brisbane, Australia; 4 Laboratory of Microbial Ecology and Technology, Ghent University, Ghent, Belgium; Texas A&M University, United States of America

## Abstract

Insulator-based dielectrophoresis can be used to manipulate biological particles, but has thus far found limited practical applications due to low sensitivity. We present linear sweep three-dimensional insulator-based dielectrophoresis as a considerably more sensitive approach for strain-level discrimination bacteria. In this work, linear sweep three-dimensional insulator-based dielectrophoresis was performed on *Pseudomonas aeruginosa* PA14 along with six isogenic mutants as well as *Streptococcus mitis* SF100 and PS344. Strain-level discrimination was achieved between these clinically important pathogens with applied electric fields below 10 V/mm. This low voltage, high sensitivity technique has potential applications in clinical diagnostics as well as microbial physiology research.

## Introduction

The cell envelope properties of bacteria play a central role in cellular function [1]. Outer membrane protein expression [2] and adhesion strength [3] can vary widely among strains of a single species. These variations in surface properties can impact clinically relevant phenotypes such as resistance to antibiotics and biofilm formation. In particular, the biofilm-forming characteristics of the Gram-negative bacterium *Pseudomonas aeruginosa* and the Gram-positive bacterium *Streptococcus mitis* have been the subject of considerable interest. *P. aeruginosa* biofilms have been extensively studied in the context of cystic fibrosis, where biofilm formation can play a key role in patient morbidity and mortality [3]–[9], as well fluid mechanics, with recent work demonstrating that particular hydrodynamic conditions can stimulate the formation of biofilm streamers [10]. Isogenic mutants of the prototype strain *P. aeruginosa* PA14 deficient or enhanced in biofilm formation have been well established [7], and their impact on infectivity verified. Similarly, *S. mitis* is an early colonizer of the oral cavity and is a pioneer constituent of dental and oropharyngeal biofilms. It is also associated with infective endocarditis, and some strains have been shown to bind directly to platelets [11], [12]. There is thus for these strains a direct relationship between their ability to form a biofilm and to infect a host. In turn, the ability to form a biofilm depends on the surface properties of the cell.

Insulator-based dielectrophoresis (iDEP) is a technique that harnesses nonlinear electrokinetic phenomena to investigate the surface properties of bacteria and other micron-scale objects [13]–[15]. iDEP utilizes the electric field gradient generated when an electric field passes through a microfluidic channel with insulating structures such as channel constrictions. If the bacterial surface is more polarizable than the surrounding solution, the cell experiences a force in the direction of the field gradient known as positive dielectrophoresis. If this force is greater than the forces on the bacterium due to electrophoresis and bulk fluid motion, the cell will be immobilized at the outlet of the constriction. This phenomena has previously been used to induce the formation of bacterial aggregates [16], but previous investigations have been hampered by low sensitivity and high applied voltages, resulting in damage to the cells and only coarse discrimination between cells with very different surface properties such as Gram-positive versus Gram-negative cells [15].

Recently, increased sensitivity for microparticle separation has been demonstrated using three-dimensional insulator-based dielectrophoresis (3DiDEP) [17]–[19]. Use of a three dimensional constriction can increase the electric field gradient and dielectrophoretic force, promoting immobilization at lower applied voltages. It has been demonstrated that 3DiDEP can be utilized to immobilize bacteria using low electric fields, ensuring cell viability and minimizing Joule heating. Previous work showed that Escherichia coli (Gram-negative) and Bacillus cereus (Gram-positive) are immobilized at different electric fields due to their cell envelope properties. Separation and discrimination of Gram-positive and Gram-negative strains has been achieved multiple times using iDEP [15], but never at such low applied electric fields. Different responses to Gram-positive and Gram-negative species are to be expected since iDEP is sensitive to the cell envelope phenotype.

Here, we demonstrate linear sweep 3DiDEP based phenotyping to measure physiological properties associated with virulence factors and biofilm formation in *P. aeruginosa* and *S. mitis*. This work represents the first demonstration that iDEP of any sort can distinguish bacteria with strain level resolution. The increased resolution is enabled by the high electric field gradient that persists in 3DiDEP systems. We analyzed wild type *P. aeruginosa* PA14, four isogenic mutants with inhibited biofilm formation capabilities (*pelA*, *pilC*, *flgK*, *cupA1*) and two mutants with enhanced biofilm formation capabilities (*pvrR*, *mucA*) (Table 1). We hypothesized that strains with enhanced biofilm formation have more polarizable cell envelopes compared to those with biofilm deficiencies and will be more easily immobilized by 3DiDEP. The endocarditis strain *S. mitis* SF100 [11] and its isogenic mutant PS344 [11], which lacks the genes for phage-encoded cell surface adhesins, were also examined using 3DiDEP. A novel measurement technique, linear sweep 3DiDEP, is introduced to test our hypothesis. We find that linear sweep 3DiDEP was able to discriminate between strains of the same species, and we also observed correlations between clinically relevant properties, cell polarizability and 3DiDEP induced cell aggregation. The results indicate that 3DiDEP based screening of bacterial pathogens has the potential for rapid, low-cost, point of care clinical diagnostics. It may also allow screening microorganisms in general towards biofilm forming phenotypes.

**Table 1:** Summary of the investigated bacterial strains.

Species	Strain	Ref.
*P. aeruginosa*	PA14	
	PA14 *cupA1*	[4]
	PA14 *flgK*	[6]
	PA14 *pilC*	[6]
	PA14 *pelA*	[8]
	PA14 *mucA*	[5]
	PA14 *pvrR*	[7]
	PA01	
*S. mitis*	SF100	[27]
	PS344	[11], [28], [29]

## Materials and Methods

### Assay and Chip Design

**Figure 1:**
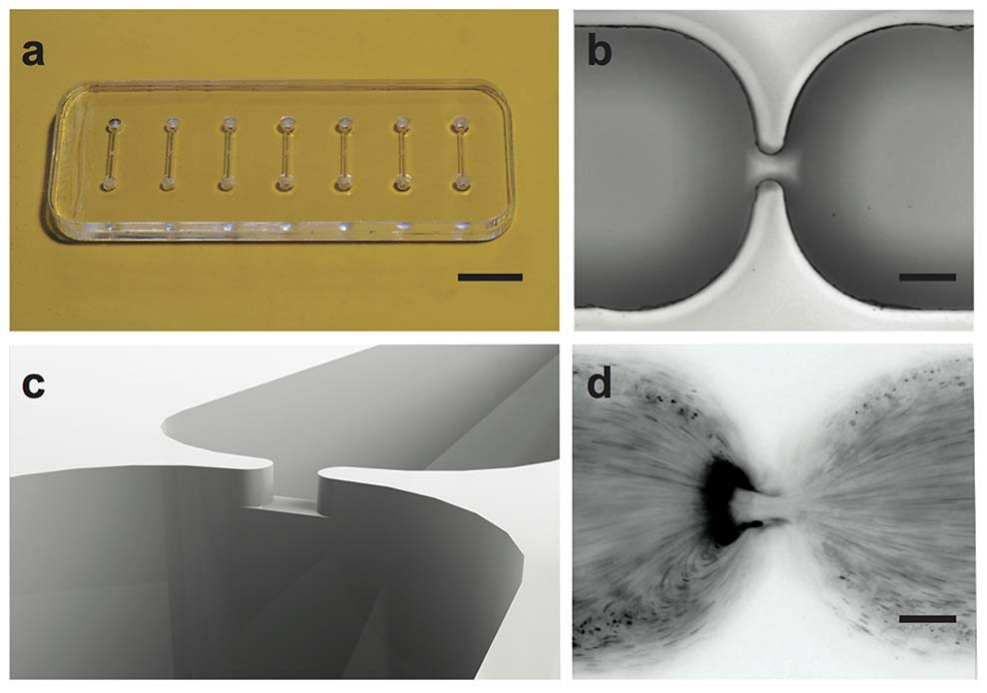
3DiDEP chip design. Photographs of microfluidic chip (a) during fabrication, and (b) micrograph of the contraction region of the channel. (c) CAD rendering of the micro channel highlighting the constriction region. (d) Sample inverted fluorescent image of *S. mitis* SF100 trapping during 3DiDEP. Scale bars are 1 cm (a), and 100 *μ*m (b and d).

To implement 3DiDEP, microfluidic channels with three-dimensional constrictions were fabricated using a precision micro-mill (Microlution 363-S, Chicago, IL) as described in detail elsewhere [17]. In the widest portion of each channel the cross sectional dimensions are 500 *μ*m×500 *μ*m while in the constriction the channel is 50 *μ*m×50 *μ*m, yielding a ratio of channel area to constriction area of 100 (Fig. 1). The high constriction ratio promotes high sensitivity iDEP at low applied voltages. Stationary growth phase test strains (Table 1) were fluorescently labeled, suspended in a neutral solution, and then injected into the chip via ports at the ends of the channel. The immobilization behavior of bacterial cells was recorded throughout the course of each experiment using a Nikon Ti-U inverted epifluorescence microscope equipped with a Photometrics Coolsnap HQ2 CCD camera.

### Device Fabrication

3DiDEP devices were machined from 1.5 mm thick poly(methyl methacrylate) (PMMA) sheets using a Microlution 363-S high precision computer numerical control mill. Reservoir ports were made using a 1.6 mm diameter endmill and microfluidic channels were made using 380 and 50 *μ*m end mills. The machined piece of PMMA was then bonded to a second blank piece using a solvent-assisted thermal bonding process to form a closed channel [20]. Fluid reservoirs were then glued to the top of the chip using two-part epoxy adhesive.

In order to ensure consistent electrokinetic properties in the device, a neutral salt solution of known conductivity and pH was used. It was prepared by adding potassium chloride to water until the solution conductivity was nearly 100 *μ*S/cm, and then titrating with potassium chloride until the pH of the solution reached 7.0, resulting in a final solution conductivity of 100 *μ*S/cm. Although the low ionic strength of the solution makes it susceptible to the formation of pH gradients during operation, the 100 *μ*L reservoirs were sufficiently large that no pH variation occurred over the course of a 100 second experiment [21]. Electroosmotic mobility of the channel was controlled by flushing the channel with 100 mM potassium hydroxide, deionized water, and solution sequentially for ten minutes each prior to every experiment.

### Bacterial Strains and Growth Conditions

The wild type *P. aeruginosa* strain PA14, as well as the *cupA1*, *pvrR* and *mucA* mutant PA14 strains were provided courtesy of Eliana Drenkard and Frederic Ausbel (Massachusetts General Hospital Simches Research Center). The PA14 mutant strains *pelA*, *pilC*, and *flgK* were provided courtesy of Sigolene Lecuyer (Harvard University). PA01 was provided by Pengfei Song and Peter Girguis (Harvard University). *P. aeruginosa* strains were grown for 16 to 18 hours at 37°C with shaking in tryptic soy broth for all stationary phase experiments. *S. mitis* strains SF100 and PS344 were provided courtesy of Paul Sullam and Ho-Seong Seo (University of California, San Francisco). *S. mitis* strains were grown for 16 to 18 hours at 37°C with shaking in Todd Hewitt broth for stationary phase experiments.

For growth phase experiments, overnight cultures were diluted 1∶100 in fresh broth (TSB for *P. aeruginosa*, and THB for *S. mitis*) and incubated at 37°C with shaking. OD_600_ was assayed every 30 minutes for the first 5 hours of growth using a spectrophotometer to determine the entry of cells into the late-lag and log phases, and then subsequently every hour of growth to monitor the progression from log to stationary phase. Culture aliquots were removed in the late-lag, mid-exponential, and stationary phases and were prepared as described below.

### Sample Preparation and Data Collection

One mL of each culture was harvested and stained with SYTO BC green-yellow fluorescent dye (1X) from Invitrogen. Stained cultures were spun at 10000×*g* to pellet cellular material, and the supernatant was discarded. Pelleted cells were washed with 1 mL of solution, and then re-suspended in 1 mL of solution. Bacterial suspensions were diluted 1∶5 in fresh solution, except lag phase cultures that were re-suspended in 200 *μ*L of solution and were not diluted. The diluted bacterial suspensions were then injected into the device. Platinum electrodes were fitted into the fluid reservoirs, and connected to an HVS-448 high voltage power supply made by Labsmith of Livermore, CA.

Two types of experiments were performed: aggregation studies at constant voltage, and linear voltage sweeps. The aggregation studies were performed by applying a constant voltage of 45 V for 100 seconds. When immobilization occurred, the bacteria accumulated just downstream of the constriction, indicating positive dielectrophoresis. After the voltage was removed, data collection continued for ten more seconds to detect the presence or absence of a stable aggregate. The bacteria were observed throughout the experiment at a rate of 1 frame per second using a Photometrics Coolsnap HQ2 CCD camera fitted to a Nikon Ti-U inverted fluorescence microscope. Image capture and voltage control was computer controlled using National Instruments Labview software. For linear sweep experiments, the voltage was ramped at a rate of 1 V/s from 10 V to 100 V to determine the minimum voltage required to induce immobilization of bacteria.

### Image Analysis

Images were captured at one frame per second throughout each experiment. In order to quantitatively compare the results, the total fluorescence intensity within the region of the chip where trapping occurs was integrated for every completed experiment, and the results were referenced to the baseline fluorescence intensity of the sample in the chip prior to applying the voltage. Between three and ten experiments were performed for each strain, and all available data were analyzed. The resulting intensity versus time data for each experiment was used to derive mean intensity and standard deviation for both the constant voltage and the linear sweep experiments. In the case of linear sweep experiments, the intensity versus time data for all available experiments were also used to generate a best fit for the two proposed parameters: threshold time and trapping rate. Error bars were calculated at the 95% confidence bounds on the fitted data. All analysis was performed using Mathworks MATLAB software package.

## Results and Discussion

### Aggregation Experiments at Constant Voltage

**Figure 2:**
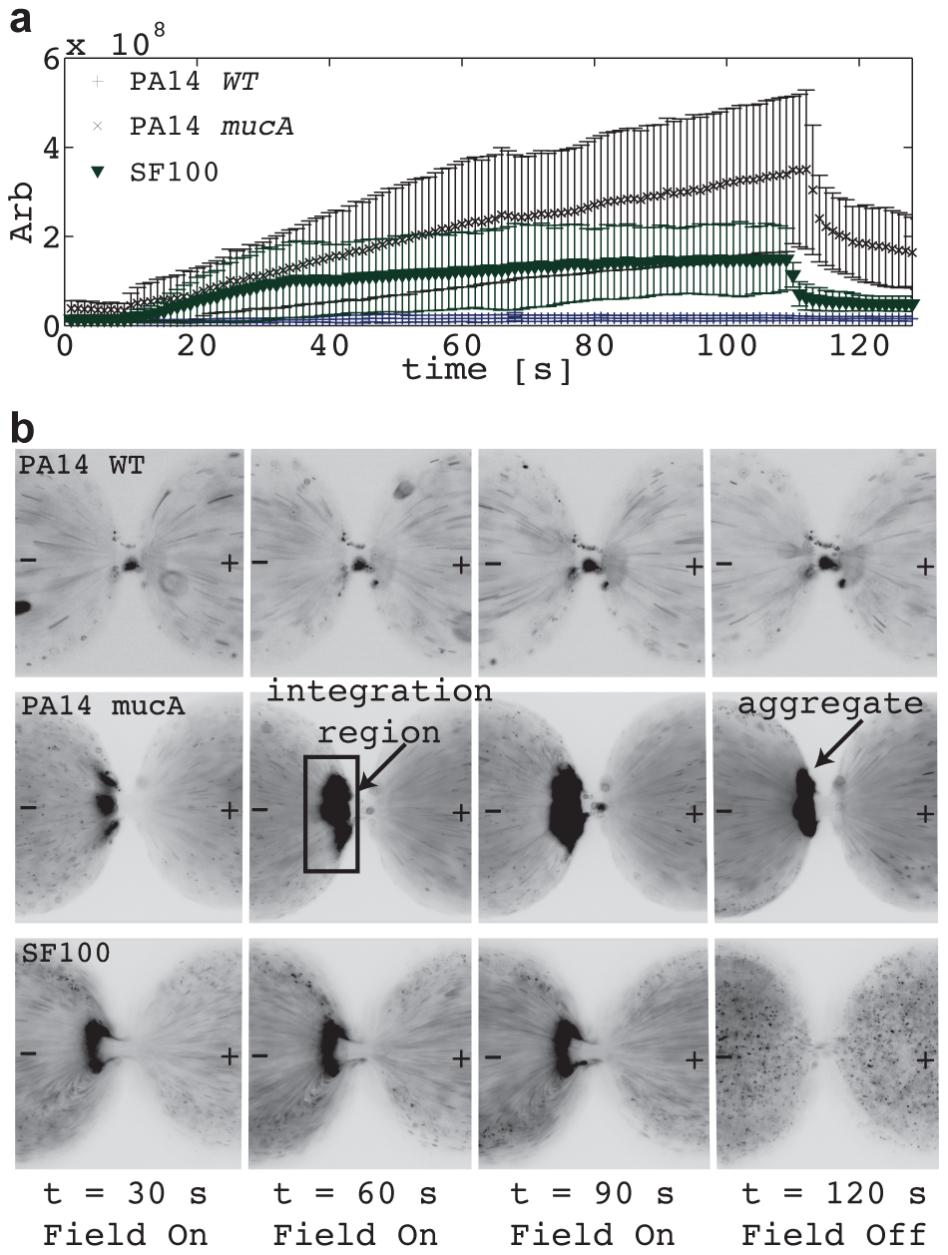
Trapping of bacteria near the constriction. Fluorescence intensity (arb) (a) and time–lapse images (b) for aggregation of *S. mitis* SF100, PA14 WT, and PA14 *mucA* when exposed to a constant voltage difference of 45 V for t = 10–110 seconds. Away from the constriction, electrophoretic force acting on the bacteria dominates over electroosmotic fluid flow and moves the bacteria from right to left towards the negative electrode. Close to the constriction, positive dielectrophoresis pulls the bacteria back into the constriction. Trapping behavior was observed in PA14 *mucA* and SF100, but not in PA14 WT, while aggregation was only observed in PA14 *mucA*. The region of integration is shown in (b).

We found that strains with a proclivity for biofilm formation assemble into tightly bound aggregates during 3DiDEP immobilization. Initially, aggregation was assessed at constant voltage using three strains; the wild-type *P. aeruginosa* PA14, the PA14 isogenic mutant *mucA*, which has been shown to have increased biofilm production [5], and *S. mitis* SF100. Mean fluorescence intensity and standard deviation near the constriction region were determined as a function of time, and are shown in Fig. 2. Recall that for a given applied electric potential, the degree of immobilization indicates the relative strength of the dielectrophoretic force experienced. Therefore the amount of cell accumulation and therefore fluorescence intensity at the constriction region is indicative of cell envelope phenotype. As shown, all three strains display significantly different levels of trapping intensity, even though PA14 and *mucA* are the same species. PA14 wild type exhibited almost no trapping (at this potential, 45 V), while *mucA* exhibited very strong trapping, indicating a more polarizable membrane. SF100 appeared to have an intermediate response. At 110 seconds, voltage was removed from the channel and the SF100 strain rapidly dispersed, returning to baseline fluorescence. Conversely, *mucA* persisted in the form of an aggregate adhered to the device, as illustrated in both the time-lapse images and the elevated fluorescence intensity after 110 seconds.

As shown by the fixed voltage experiments, trapped bacteria either dispersed rapidly or remained aggregated after the voltage was removed (Fig. 2). Wild type PA14 and PA01 both formed aggregates, as evidenced by the residual fluorescence intensity after the voltage was shut off, as did the enhanced biofilm strains *mucA* and *pvrR*. Conversely, all biofilm-deficient strains, including *pelA*, dispersed when the voltage was turned off, suggesting a strong correlation between aggregation and biofilm forming phenotypes. Note that after the field is removed at 100 s the bacteria can be subject to pressure driven flow resulting from height differences in the outlet reservoirs. The height differences result from electroosmotic flow in the channel during operation. This is the reason for the streaks of wild type PA14 and PA14 *mucA* at 120 s in Fig. 2b.

### Linear Sweep iDEP

**Figure 3:**
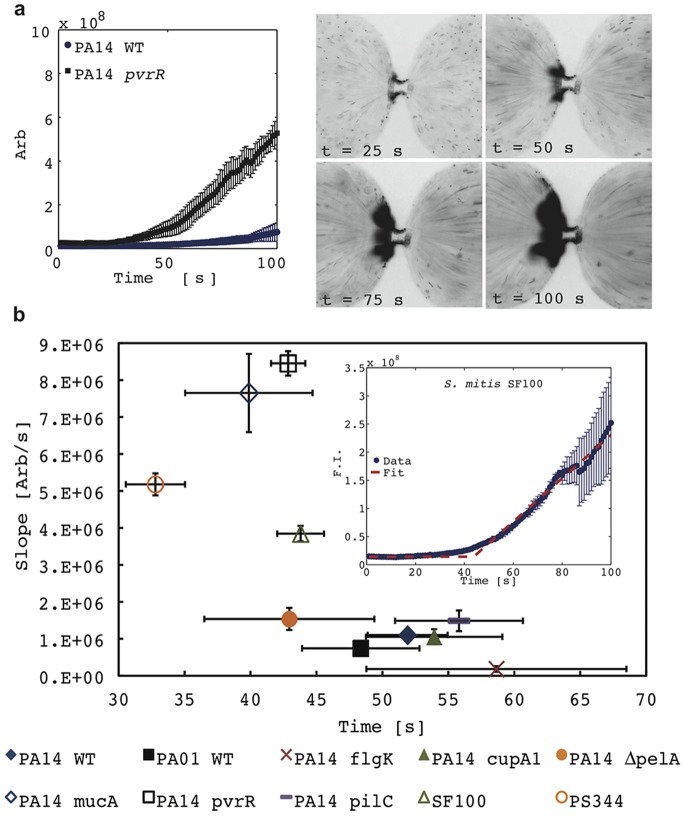
Analysis of key trapping parameters. Integrated fluorescence intensity of PA14 WT and PA14 *pvrR* as a function of time, with representative inverted fluorescent images of PA14 *pvrR* (a). Voltage was swept at 1 V/s for 100 seconds (e.g. 50 s corresponds to 50 V applied). The response can be characterized by the time at which trapping can first be observed and the rate at which bacteria accumulate. Trapping initiation and bacteria accumulation rate for all ten strains investigated (b). Initial trapping and cell accumulation rate are fitted as shown in the inset. *pvrR*, *mucA*, and *pelA* demonstrate earlier and stronger responses than PA14 WT, while *flgK*, *pilC*, and *cupA1* exhibit later and weaker responses. Accumulation rates for S. mitis SF100 and PS344 are similar, but PS344 traps earlier (i.e. at a lower voltage).

**Figure 4:**
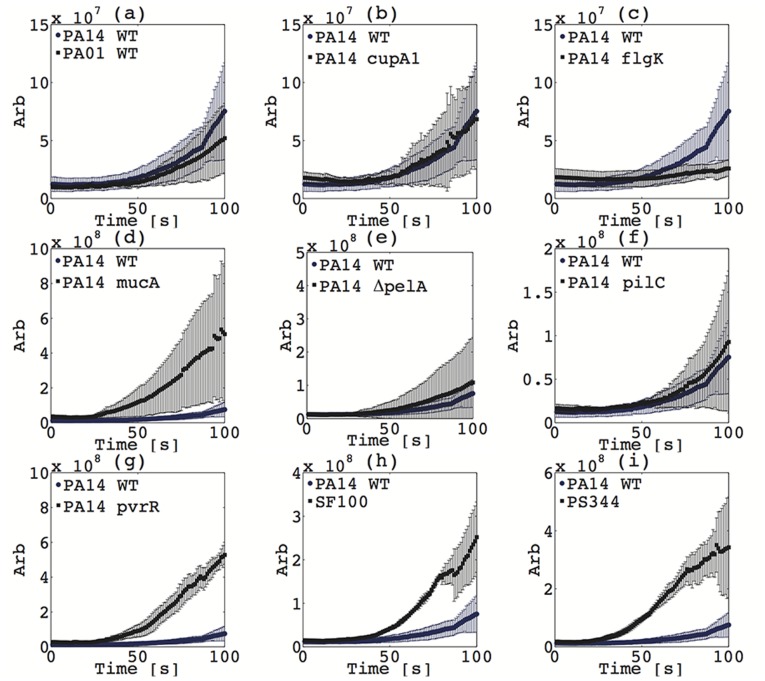
Linear sweep trapping intensity varies across strains of bacteria. Fluorescence intensity as a function of time as voltage was swept at 1/s for *P. aeruginosa* PA01 WT (a), *cupA1* (b), *flgK* (c), *mucA* (d), *pelA* (e), *pilC* (f), *pvrR* (g), *S. mitis* SF100 (h), and PS344 (i) compared to *P. aeruginosa* PA14 WT.

The ability to rapidly discriminate between strains is valuable for certain applications, but the binary parameters of trapping and aggregation are insufficient to characterize a heterogeneous population. Here we perform a technique denoted linear sweep iDEP in order to characterize cell envelope polarizability. In linear sweep iDEP, voltage is increased linearly (here at a rate of one volt per second), thus rapidly providing information about trapping at a range of voltages. As stated above, for a given cell there is a particular electric field where dielectrophoretic forces at the constriction exceed electrophoresis and the background electroosmosis. Sweeping the applied electric field is an efficient method to determine the minimum electric field where immobilization occurs. The minimum electric field for immobilization varies for different strains since it’s a strong function of cell envelope phenotype. Note that a similar technique has been previously employed to determine the minimum potential required for iDEP immobilization of bacteria [15]. In this study, linear sweep iDEP was conducted in triplicate (at a minimum) for each strain listed in Table 1, and the average and standard deviation in fluorescence intensity were calculated. The voltage is swept from 10 V to 100 V at a rate of 1 V/s and beginning 10 s after the experiment is initiated. Thus, 10 s corresponds to 10 V applied, 30 s corresponds to 30 V applied, etc. The first 100 seconds of the experiments are shown in Fig. 3a for PA14 WT and PA14 *pvrR*. Fluorescence intensity data for the other strains is shown in Fig. 4.

The trapping strength *α* in iDEP devices is defined as the ratio of the dielectrophoretic force to the electrophoretic and electroosmotic effects on the particle or cell. Trapping strength scales linearly with applied voltage, *V_0_*, and for a particle of radius *a* and electrophoretic mobility 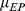 suspended in a media of electroosmotic mobility 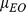
and relative permittivity 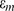 in a channel of length *L*, square cross-sectional width *w* and constriction ratio 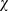, 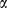 can be approximated as [17]:
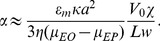
(1)


Here, 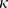 is the geometry dependent Clausius-Mossotti factor, which expresses the relative polarizability of the suspended particles relative to the media. It is therefore reasonable to fit the fluorescence intensity versus time data for linear sweep iDEP in terms of a minimum voltage at which trapping appears and the rate at which cells accumulate near the constriction, with these parameters representing bacterial polarizability. A best fit was performed for each strain of bacteria, with the resulting trapping initiation times and accumulation rates plotted (Fig. 3b).

### Dielectrophoretic Cell Envelope Phenotyping

Nearly all isogenic mutants of PA14 tested demonstrated trapping behavior that was representative of their ability to form biofilms. The *mucA* and *pvrR* strains demonstrated substantially enhanced trapping, roughly eight times stronger than wild type PA 14 with initial trapping occurring approximately ten volts lower (i.e. ten seconds earlier). This is consistent with their previously demonstrated ability for increased biofilm formation [7], [22]. Discrimination of *mucA* mutants is of particular clinical interest, as *mucA* is the most common mutation in the conversion of cystic fibrosis strains of *P. aeruginosa* to its mucoid phenotype [5]. This shift to mucoidy can often be indicative of more severe disease and poorer clinical outcomes [23]. The biofilm-deficient strains *cupA1*, *flgK*, and *pilC* all trapped slightly later than wild type, with *flgK* exhibiting the weakest trapping. The differences in electric potential required for trapping suggest differences in cell envelope phenotype, which is to be expected given that the various mutations target aspects of biofilm formation. Observed trapping is a result of a force balance between electrophoresis and dielectrophoresis, so this work does not address whether the cell envelope phenotype variation is due to changes in polarizability or electrophoretic mobility. However, previous work has shown that electrophoretic mobility varies very little within a given species, and suggests that the dominant mechanism of discrimination is polarizability [24]. The *cupA1* and *flgK* mutants are defective in biofilm initiation while the *pilC* mutant exhibits reduced late stage biofilm development [4]. The *flgK* mutant cannot form a flagellum, which may account for the additional deficit in trapping via alteration of cell surface properties. Only *pelA*, the mutant unable to produce the biofilm encapsulating protein PelA, trapped slightly earlier and stronger than PA14 contrary to expected behavior, although still being readily distinguishable from the wild type strain (Fig. 3b).

*S. mitis* SF100 and PS344 demonstrated unique trapping and aggregation behavior, which distinguished them from the *P. aeruginosa* strains as well as from each other, as shown in Fig. 2. Both SF100 and PS344 trapped earlier and stronger than wild type PA14, though slightly less so than *mucA* or *pvrR*. PS344, the isogenic mutant of SF100 lacking genes for the phage-encoded adhesins PblA and PblB, exhibited greater trapping than SF100. The PblA and PblB proteins bind to choline residues on the surface of microbial cells and may alter the cell-surface properties of *S. mitis* [11]. Ablation of PblA and PblB has been shown to decrease *S. mitis* virulence in an animal model of endocarditis, inhibiting the ability of *S. mitis* to bind platelets and form infective vegetations [11]. The differential trapping behavior of SF100 and PS344 suggests that linear sweep 3DiDEP can be used to identify *S. mitis* strains with cell-surface adhesins attributed to increased virulence.

Previous implementations of iDEP have been used to sort based on obvious traits such as Gram-positive versus Gram-negative bacteria, live versus dead bacteria, and in some cases members of the same genus [15], [25]. We present high sensitivity 3DiDEP as a rapid and inherently low cost technique for discriminating between specific bacterial strains or isogenic mutants. We demonstrate that 3DiDEP enables resolution of strains of *P. aeruginosa* PA14 and *S. mitis* using trapping intensity and aggregate formation as the phenotypic observables. Furthermore, it is possible to discriminate between specific mutations within a particular species using two measured parameters, trapping intensity and aggregation. The direct observation of phenotype, rather than genotype, allows for rapid discrimination and in the future could lead to point of care applications of microbe identification.

## Conclusion

3DiDEP phenotyping should be suitable for a range of applications. For example, despite the increasing prevalence of antibiotic resistant strains it is common practice for physicians to prescribe antibiotics without confirming the diagnosis. This is because existing techniques such as sequencing and rRNA fingerprinting are either too slow or too expensive to be used as a diagnostic tool for many pathogens [26]. The apparent correlation between biofilm formation and aggregation during 3DiDEP tests is compelling evidence that 3DiDEP has potential as a rapid diagnostic tool that is durable to locus mutations, which can disrupt genotypic methods such as PCR. Future experiments will further calibrate this approach by testing heterogeneous cultures comprised of a diverse collection of microorganisms. In order for this technique to truly be low cost, it must be able to operate without additional laboratory equipment such as a fluorescence microscope. This could be accomplished by measuring channel impedance during the experiment as a proxy for fluorescence intensity.
